# Financial toxicity, family resilience and symptom burden in liver cancer patients: a cross-sectional study

**DOI:** 10.3389/fpsyg.2025.1747893

**Published:** 2026-01-29

**Authors:** Jie Zou, Qin Luo, Yanhong Yang, Na Li, Jin Zhang, Zhaoli Zhang

**Affiliations:** 1 Hepatobiliary Pancreatic Cancer Center, Chongqing University Cancer Hospital, Chongqing, China; 2 Department of Nursing, Chongqing University Cancer Hospital, Chongqing, China

**Keywords:** cross-sectional study, family resilience, financial toxicity, liver cancer, symptom burden

## Abstract

**Background:**

Financial toxicity has emerged as a critical concern in oncology care, reflecting the severe economic and psychological distress patients experience due to treatment costs. While socioeconomic factors are known contributors, the potential protective role of psychosocial assets, such as family resilience, remains underexplored. This study aimed to investigate the prevalence and predictors of financial toxicity among liver cancer patients in China, with a specific focus on the roles of family resilience, psychological distress, and symptom burden.

**Methods:**

Participants were required to complete comprehensive scores for financial toxicity based on the patient-reported outcome measures (COST-PROM), the Chinese version of family resilience assessment scale (FRAS-C), the validated Edmonton symptom assessment scale (ESAS) and the patient health questionnaire-4 (PHQ-4). Data of demographic and clinical characteristics were also collected. Descriptive statistics, correlation analyses, and hierarchical multiple linear regression were used to analyze the data. This study adhered to the STROBE guidelines.

**Results:**

The mean financial toxicity score was 20.36 ± 3.95. Hierarchical regression revealed a significant model (R^2^ = 0.601, *p* < 0.001) for financial toxicity. Key risk factors included psychological distress (β = −0.594, *p* < 0.001), advanced disease stage (β = −0.339, *p* < 0.001), and recent diagnosis (β = −0.325, *p* < 0.001). Conversely, higher family resilience was a significant protective factor (β = 0.207, *p* < 0.001). A counterintuitive finding was that greater symptom burden was associated with higher FT scores (indicating less severe FT) (β = 0.159, *p* = 0.002). Several socioeconomic factors (e.g., lower income, living alone) were also significant predictors.

**Conclusion:**

Financial toxicity is prevalent among Chinese liver cancer patients and is influenced by a complex interplay of economic, clinical, and psychosocial factors. The findings underscore that family resilience is a key buffer against financial distress, while psychological distress is a powerful amplifier. This study highlights the necessity for integrated supportive care interventions that address not only the economic but also the psychological and familial dimensions of cancer care to effectively mitigate financial toxicity.

## 1 Introduction

Liver cancer remains a formidable global health challenge, characterized by its high incidence, substantial mortality, and significant economic burden (Sung et al., 2021). According to Chinese cancer 2022 statistics, liver cancer ranks as the second most prevalent cause of cancer-related deaths and the fourth most common cancer, presenting a significant public health risk (Zheng et al., 2024). The financial expenditure associated with diagnosing, treating, and managing liver cancer and its complications is considerably higher than that for many other solid tumors, placing an immense and often unsustainable burden on healthcare systems, families, and individual patients (Karim et al., 2023). Despite improvements in insurance coverage, reimbursement rates for many expensive targeted therapies and immunotherapies remain partial, shifting a significant financial responsibility onto patients and their families (Yamamoto et al., 2024). The economic impact extends beyond direct medical costs to include indirect costs such as lost productivity, travel expenses for treatment, and the need for informal caregiving (Teli et al., 2025). For many households, a liver cancer diagnosis can precipitate a rapid descent into financial catastrophe, leading to treatment non-adherence, diminished quality of life, and worse survival outcomes.

In recent years, the concept of financial toxicity (FT) has emerged as a critical patient-reported outcome in oncology. First coined by Zafar et al. (2013), FT captures the multidimensional financial distress and associated psychological burden experienced by patients as a direct result of cancer treatment (Ehsan et al., 2023). This distress extends beyond direct medical expenses to encompass indirect costs such as lost income, travel for treatment, and informal caregiving (Teli et al., 2025). For patients with liver cancer, FT is especially acute due to the need for prolonged, often lifelong surveillance, combined with high-cost therapies that may only be partially covered by insurance (Blinder, 2022; Yamamoto et al., 2024). Furthermore, older age has been identified as an independent risk factor for severe FT in cancer populations, compounding the economic vulnerability of patients. Studies indicate that a significant proportion of liver cancer patients experience FT, which in turn is strongly associated with treatment delays, non-adherence, reduced quality of life, and potentially worse survival outcomes (Aby and Ufere, 2023; Zhang Y. et al., 2025). Importantly, liver cancer often affects populations with pre-existing socioeconomic vulnerabilities–such as chronic hepatitis B or C carriers–further exacerbating health disparities and complicating timely care (Blanter et al., 2022). In the Chinese context, such disparities are pronounced, with evidence indicating that rural residence is an independent risk factor for more severe FT among cancer patients, likely due to inequalities in insurance coverage, income, and access to healthcare resources (Xu et al., 2022).

Within this context of clinical and economic adversity, the family unit in China typically serves as the primary source of support for patients. This underscores the potential relevance of family resilience, a construct grounded in Walsh’s family resilience framework, which emphasizes a family’s capacity to withstand, adapt, and grow from crises through shared belief systems, flexible organizational patterns, and open communication processes (Walsh, 1998; Vladislav et al., 2024). Rather than focusing solely on family deficits, this strengths-based perspective highlights how families mobilize internal and external resources to navigate challenges. In the setting of liver cancer–a disease that often occurs in patients with complex pre-existing conditions like cirrhosis–family resilience may manifest through collective financial resource pooling, coordinated caregiving, emotional bolstering during health declines, and effective collaboration with healthcare providers (Cui et al., 2023). While the broader protective role of family support in psychosocial oncology is well recognized, the specific mechanisms through which family resilience might buffer against FT remain underexplored, particularly in cultural settings like China where familial interdependence is deeply embedded.

Liver cancer and its treatments are also associated with a severe and multifaceted symptom burden, including fatigue, pain, nausea, appetite loss, and cognitive changes, all of which significantly impair daily functioning and quality of life (Zhang W. Z. et al., 2025). The relationship between symptom burden and FT is complex and likely bidirectional. High symptom burden may increase healthcare utilization and costs, thereby intensifying FT. Conversely, financial worry and distress may exacerbate the perception of symptoms and undermine self-management. However, this interplay is not fully understood, and some evidence–including paradoxical observations from preliminary data–suggests that overwhelming physical suffering may sometimes temporarily overshadow financial concerns, a nuance that merits further investigation (Chan et al., 2019).

Despite the clear clinical and economic significance of FT in liver cancer, several important gaps persist in the literature. First, most studies have focused on demographic or clinical predictors of FT, with less attention paid to modifiable psychosocial and family-level factors. Second, while family resilience has been studied in various health contexts, its specific role in mitigating economic distress in oncology–and particularly in liver cancer–remains empirically underexamined. Third, few investigations have simultaneously examined the interrelationships among FT, family resilience, and symptom burden within a unified framework, limiting a holistic understanding of how these factors interact to shape patient outcomes. By illuminating these complex interrelationships, this research aims to contribute to the development of more nuanced, effective, and culturally sensitive interventions that address not only the clinical but also the financial and familial dimensions of liver cancer care, ultimately aiming to alleviate suffering and improve outcomes for patients and their families.

## 2 Materials and methods

### 2.1 Design and participants

We conducted an observational cross-sectional study in tertiary hospitals in Chongqing, China. We recruited patients using consecutive sampling from January to May 2025. Inclusion criteria: (1) hospitalized patients with a confirmed diagnosis of primary liver cancer; (2) aged 18 years or older; (3) clinically stable with a Karnofsky Performance Status (KPS) score ≥ 60; (4) voluntary participation. There were two exclusion criteria: (1) comorbidities with other malignancies or life-threatening diseases; and (2) History of psychiatric disorders, cognitive impairments (e.g., severe deficits in comprehension, memory, or orientation), or significant auditory/visual impairments. An *a priori* power analysis was performed using G*Power software (Faul et al., 2007). For a linear multiple regression model (fixed model, R^2^ deviation from zero) with 12 predictors, an alpha (α) level of 0.05, and a statistical power (1−β) of 0.80, a minimum sample size of 113 participants was required to detect a medium effect size (f^2^ = 0.15). To account for an estimated 20% rate of invalid questionnaires, the target sample size was inflated to 142. Our final sample of 495 participants far exceeds this requirement, ensuring adequate power for the analyses. This study adhered to the STROBE guidelines (Supplementary material).

### 2.2 Measures

#### 2.2.1 General information questionnaire

A self-designed demographic questionnaire was employed to collect participants’ baseline characteristics, including age, gender, education level, marital status, household income, employment status, and clinical characteristics (e.g., treatment methods). This instrument has been pretested for clarity and appropriateness in the target population during the pilot phase of the study.

#### 2.2.2 Chinese version of comprehensive scores for financial toxicity based on the patient-reported outcome measures (COST-PROM)

Financial toxicity was assessed using the Chinese version of the Comprehensive Score for Financial Toxicity based on Patient-Reported Outcome Measures (Luo, 2023). This 11-item instrument covers three dimensions: financial expenditure, financial resources, and psychosocial impact. Patients rated each item based on their experience over the past week on a 5-point Likert scale from 0 (“not at all”) to 4 (“very much”). Items 2, 3, 4, 5, 8, 9, and 10 are reverse-scored. The total score ranges from 0 to 44, with lower scores indicating greater financial toxicity. Based on established cut-offs (Luo, 2023), scores are categorized as: no FT (≥24), mild (17–23), moderate (13–16), or severe (≤12). The scale demonstrated good reliability, with a Cronbach’s α of 0.874 and a test-retest reliability of 0.972, indicating satisfactory psychometric properties.

#### 2.2.3 Chinese version of family resilience assessment scale (FRAS-C)

Family resilience was measured using the 32-item FRAS-C, a culturally adapted and simplified self-report instrument derived from the original family resilience assessment scale developed by Sixbey (2008). The FRAS-C was linguistically validated and psychometrically tested by Li et al. (2016) for use in Chinese populations. The scale comprises three dimensions: utilization of social resources (3 items), maintaining a positive outlook (6 items) and family communication and problem-solving (23 items). Each item is rated on a 4-point Likert scale (1 = strongly disagree to 4 = strongly agree). The total score ranges from 32 to 128, with higher scores indicating stronger family resilience. The FRAS-C has demonstrated good internal consistency in previous validation studies (Cronbach’s α = 0.89∼0.92 for subscales and 0.94 for the total scale) (Wang X. et al., 2024; Wang Y. et al., 2024). In the current study, the Cronbach’s α for the total scale was 0.93.

#### 2.2.4 Symptom burden

Using the validated Edmonton Symptom Assessment Scale (ESAS) (Bruera et al., 1991) and the Patient Health Questionnaire-4 (PHQ-4) (Kroenke et al., 2009), we evaluated the baseline physical and psychological symptom load of the patients. Pain, exhaustion, drowsiness, nausea, appetite, dyspnea, depression, anxiety, well-being, insomnia, constipation, and diarrhea are among the symptoms that the ESAS scale evaluates. A 0∼10 scale is used to rate each particular symptom, with 0 denoting the absence of the symptom and 10 denoting its severe severity. We employed the PHQ-4, a four-item instrument with two-item subscales, to more precisely assess symptoms of anxiety and depression; higher scores indicate greater severity of these symptoms (Kroenke et al., 2009). The scale comprises two subscales: anxiety (e.g., “feeling nervous, anxious, or on edge”) and depression (e.g., “little interest or pleasure in doing things”), each with two items rated on a 4-point Likert scale (0 = not at all to 3 = nearly every day). The PHQ-4 yields a total score ranging from 0 to 12, where higher scores indicate a greater symptom burden. Based on the total score, the severity of symptoms is categorized as none (0–2), mild (3–5), moderate (6–8), or severe (9–12). The PHQ-4 demonstrates strong validity and reliability (Cronbach’s α = 0.82–0.85) (Caycho-Rodríguez et al., 2024). In this study, Cronbach’s α of ESAS and PHQ-4 was 0.84 and 0.89 respectively.

### 2.3 Data collection

Three research assistants were trained extensively on the specifics of the study before data collection began. A unified guidance was used to meet with subjects and provide them with questionnaires. In the ward, face-to-face surveys were conducted, which included both an introduction to the project and the main questionnaire. This cross-sectional study’s purpose, risks, and benefits were explained to each participant and the hospital director prior to conducting the study.

### 2.4 Ethical considerations

This study was conducted in full compliance with international ethical standards and was approved by the institutional review board of Chongqing university cancer hospital [No. CZLL (2025) - (035) - 001]. Eligibility screening was conducted by two independent investigators before recruitment. Qualified candidates received comprehensive verbal and written explanations regarding the study protocol prior to providing written consent for participation. All participants provided written informed consent after receiving detailed explanations regarding the study objectives, procedures, potential risks, and benefits.

### 2.5 Statistical analysis

Excel was used to create the questionnaire database, and data was analyzed using SPSS (version 22.0). Categorical variables were described using frequencies and percentages, while continuous variables conforming to normal distribution were presented as mean ± standard deviation. Group comparisons were performed using *t*-tests or one-way ANOVA. Pearson correlation analysis was employed to examine relationships between FT, family resilience, psychological distress, and symptom burden. Hierarchical multiple linear regression was used to identify factors influencing financial toxicity. Collinearity among independent variables was assessed by examining the variance inflation factor (VIF) and tolerance; a VIF < 5 and tolerance > 0.2 indicated no substantial multicollinearity (Field, 2009). A two-tailed *P*-value < 0.05 was considered statistically significant.

## 3 Results

### 3.1 Participant characteristics and differences in FT

A total of 495 participants were included in this study. The demographic and clinical characteristics of the sample, along with their differences in FT, are presented in Table 1. The sample was nearly evenly distributed by gender, with 244 (49.3%) males and 251 (50.7%) females. The majority of participants were aged 51 years or older (78.4%), and most had an educational level of senior high school or below (89.7%). A significant proportion were married (83.4%) and had two children (65.5%). Approximately half of the participants resided in rural areas (50.7%). FT scores differed significantly across various demographic and clinical characteristics.

**TABLE 1:** Characteristics of the participants and demographic differences in FT.

Variables	*n* (%)	Mean ± SD	*t/F*	*p*
Gender			5.462	<0.001
Male	244 (49.3%)	19.16 ± 0.26		
Female	251 (50.7%)	21.51 ± 0.22		
Age (years)			12.783	<0.001
18∼35	18 (3.6%)	20.54 ± 0.19		
36∼50	89 (18.0%)	19.77 ± 0.53		
≥51	388 (78.4%)	19.11 ± 0.76		
Education level			5.203	0.006
≤Junior high school below	182 (36.8%)	19.80 ± 0.31		
Senior high school	262 (52.9%)	20.40 ± 0.24		
College	51 (10.3%)	22.09 ± 0.32		
Marital status			2.571	0.077
Unmarried	10 (2.0%)	18.00 ± 0.04		
Married	413 (83.4%)	20.64 ± 0.19		
Divorced/ separated	72 (14.5%)	19.03 ± 0.49		
Childbearing			0.692	0.501
No	20 (4.0%)	21.00 ± 0.69		
One child	151 (30.5%)	20.60 ± 0.32		
Two children	324 (65.5%)	20.20 ± 0.22		
Residence			−0.723	0.574
Urban	244 (49.3%)	19.33 ± 0.22		
Rural	251 (50.7%)	20.39 ± 0.31		
Living conditions			8.277	<0.001
With spouse	200 (40.4%)	20.21 ± 0.21		
With children	104 (21.0%)	20.45 ± 0.35		
With spouse and children	110 (22.2%)	22.72 ± 0.39		
Alone/other	81 (16.4%)	17.39 ± 0.46		
Occupation			2.554	0.079
Unemployed	274 (55.4%)	19.60 ± 0.21		
Employed	80 (16.2%)	23.39 ± 0.44		
Retired/on leave of service	141 (28.5%)	20.09 ± 0.33		
Monthly income (CNY^a^)			16.623	<0.001
<4500	150 (30.3%)	18.47 ± 0.32		
4500∼7000	192 (38.8%)	21.47 ± 0.26		
>7000	153 (30.9%)	20.80 ± 0.31		
Time of diagnosis (month)			10.521	<0.001
<6	263 (53.1%)	20.36 ± 0.25		
6–12	71 (14.3%)	20.26 ± 0.35		
>12	161 (32.5%)	20.39 ± 0.33		
The number of hospitalizations (times)			1.392	0.250
1	91 (18.4%)	20.85 ± 0.33		
2	91 (18.4%)	20.95 ± 0.34		
≥3	313 (63.4%)	20.03 ± 0.32		
Staging of disease			13.423	<0.001
I	51 (10.3%)	23.16 ± 0.37		
II	152 (30.7%)	20.98 ± 0.32		
III	142 (28.7%)	19.33 ± 0.36		
IV	150 (30.3%)	19.74 ± 0.28		
Treatment methods			11.772	<0.001
Chemotherapy	101 (20.4%)	20.07 ± 0.64		
Surgery	121 (24.4%)	21.82 ± 0.59		
Immunotherapy + targeted therapy	163 (32.9%)	20.61 ± 0.54		
Others	110 (22.2%)	22.55 ± 0.73		
Concurrent other diseases (kinds)			23.864	<0.001
None	332 (67.1%)	20.25 ± 0.22		
1∼2	133 (26.9%)	20.02 ± 0.31		
≥3	30 (6.1%)	23.00 ± 0.66		

SD, standard deviation.

*^a^*CNY China Yuan, US$ 1.00 = ¥ 7.13.

### 3.2 Descriptive statistics of key variables

As presented in Table 2, the FT score was 20.36 ± 3.95. The family resilience score was 99.44 ± 9.33. The average score for psychological distress was 6.27 ± 2.51, and the symptom burden score was 25.39 ± 12.74. FT was negatively correlated with family resilience (*r* = −0.662, *p* < 0.01) and positively correlated with psychological distress (*r* = 0.426, *p* < 0.01) and symptom burden (*r* = 0.118, *p* < 0.01). Family resilience was also negatively correlated with psychological distress (*r* = −0.300, *p* < 0.01) and symptom burden (*r* = −0.282, *p* < 0.01).

**TABLE 2:** Descriptive statistics and correlations among variables.

Variables	Ranges	Mean ± SD	1	2	3	4
1. Financial toxicity	0∼44	20.36 ± 3.95	1			
2. Family resilience	32∼128	99.44 ± 9.33	−0.662**	1		
3. Psychologic distress	0∼12	6.27 ± 2.51	0.426**	−0.300**	1	
4. Symptom	0∼100	25.39 ± 12.74	0.118**	−0.282**	0.414**	1

Pearson test. **p < 0.010.

### 3.3 Multivariate regression analysis of FT

A hierarchical multiple linear regression analysis was performed to identify factors independently associated with FT (Table 3). The final model was statistically significant (*F* = 28.980, *p* < 0.001), explaining 60.1% of the variance in FT scores (R^2^ = 0.601). All VIF values are <5 and all Tolerance values are >0.2, indicating no significant multicollinearity. Several demographic, clinical, and psychosocial factors emerged as significant predictors of FT. Among demographic factors, lower educational level was associated with higher FT (β = −0.090, *p* = 0.033). Relative to patients in the highest monthly income bracket (>7000 CNY), those with middle (4500–7000 CNY; β = −0.205, *p* < 0.001) or low income (<4500 CNY; β = −0.154, *p* = 0.008) reported significantly lower FT scores, indicating more severe financial toxicity. Compared to living with a spouse, living with children was associated with higher FT scores (β = 0.178, *p* < 0.001), suggesting a protective effect. In contrast, living alone or in other arrangements was associated with significantly lower FT scores (β = −0.184, *p* < 0.001), identifying it as a risk factor for more severe financial toxicity. Older age was associated with lower FT (β = −0.461, *p* < 0.001). For clinical factors, a more recent time since diagnosis (β = −0.325, *p* < 0.001) and the presence of a greater number of comorbidities (β = −0.185, *p* < 0.001) predicted higher FT. Compared to patients with Stage I disease (reference), those with Stage II (β = 0.161, *p* = 0.020) reported significantly higher FT scores (i.e., less severe FT), while those with Stage III disease (β = −0.339, *p* < 0.001) reported significantly lower FT scores, indicating more severe FT. Crucially, among psychosocial factors, higher family resilience was a significant protective factor (β = 0.207, *p* < 0.001), while greater psychological distress was the strongest predictor of worse FT in the model (β = −0.594, *p* < 0.001). A counterintuitive finding was that higher symptom burden predicted less severe FT (β = 0.159, *p* = 0.002). Other variables, including gender and treatment modalities, did not show statistically significant associations in the final model. A notable methodological observation is the change in association for psychological distress between bivariate and multivariate analyses. It showed a positive bivariate correlation with the FT score (*r* = 0.426, Table 2) but emerged as a strong independent risk factor (negative β) in the regression model. This pattern is consistent with a statistical suppressor effect, where the initial positive link may have been confounded by its correlation with other protective factors (e.g., socioeconomic resources). Controlling for these covariates in the multivariate model unmasked its distinct adverse effect on FT. The relationship between symptom burden and FT, while also shifting between analyses, ultimately showed a protective association (positive β) in the final model.

**TABLE 3:** Hierarchical regression analysis for predicting FT.

Variable	B	SE	β	t	*p*	95% CI	Tolerance	VIF
Constant	29.562	5.132	–	5.760	<0.001	(19.488, 39.636)		
Age	−7.891	1.347	−0.461	−5.857	<0.001	(−10.535, −5.247)	0.82	1.22
Education	−2.077	0.973	−0.090	−2.134	0.033	(−3.986, −0.168)	0.88	1.14
**Living status**								
(Code 2)	3.079	0.847	0.178	3.634	<0.001	(1.417, 4.741)	0.75	1.33
(Code 3)	−3.110	0.689	−0.184	−4.514	<0.001	(−4.461, −1.759)	0.78	1.28
**Monthly income**								
(Code 2)	−3.011	0.700	−0.205	−4.304	<0.001	(−4.384, −1.638)	0.69	1.45
(Code 3)	−2.356	0.885	−0.154	−2.663	0.008	(−4.092, −0.620)	0.72	1.39
Time of diagnosis	−6.526	0.967	−0.325	−6.751	<0.001	(−8.422, −4.630)	0.85	1.18
**Disease stage**								
(Code 2)	2.457	1.050	0.161	2.339	0.020	(0.395, 4.519)	0.80	1.25
(Code 3)	−5.418	0.958	−0.339	−5.658	<0.001	(−7.298, −3.538)	0.77	1.30
Comorbidity	−5.439	1.300	−0.185	−4.184	<0.001	(−7.990, −2.888)	0.90	1.11
Family resilience	3.712	0.720	0.207	5.157	<0.001	(2.299, 5.125)	0.86	1.16
PHQ-4	−1.676	0.127	−0.594	−13.186	<0.001	(−1.925, −1.427)	0.89	1.12
Symptom burden	0.063	0.020	0.159	3.102	0.002	(0.023, 0.103)	0.91	1.10

B, unstandardized coefficient; β (Beta), standardized coefficient; SE, standard error; CI, confidence interval; VIF, variance inflation factor.

Categorical variables were coded as follows: Living Status (Reference: Code 1: with spouse; Code 2: with children; Code 3: alone/other); Monthly Income (Reference: Code 1: >7000 CNY; Code 2: 4500–7000 CNY; Code 3: <4500 CNY); Disease Stage (Reference: Code 1: Stage I; Code 2: Stage II; Code 3: Stage III).

The overall model was statistically significant, *F* = 28.980, *p* < 0.001, accounting for 60.1% of the variance (R^2^ = 0.601).

## 4 Discussion

To our knowledge, this is the first cross-sectional study to comprehensively examine the interplay between FT, family resilience, psychological distress, and symptom burden in a Chinese liver cancer population. Our findings reveal that FT extends beyond a simple financial calculation to represent a complex biopsychosocial syndrome, deeply influenced by the patient’s psychosocial context. The regression model explained a substantial proportion of the variance in FT (R^2^ = 60.1%), highlighting the critical importance of integrating these non-clinical factors into cancer care models. While socioeconomic and clinical factors establish the baseline risk, psychological distress and family resilience emerged as pivotal forces that respectively amplify or buffer the subjective experience of financial hardship.

### 4.1 The current state of financial toxicity

Our participants yielded a mean COST-PROM score of 20.36 ± 3.95, indicating mild FT among cancer patients. Our mean score is higher than the 17.98 reported among cancer patients in China (Qiu et al., 2023), where high out-of-pocket expenditures and coping strategies such as borrowing money were directly linked to more severe financial toxicity. This contrast suggests that the insurance coverage or social support mechanisms available to our participants may offer a greater, albeit partial, buffer against direct medical costs. On the other hand, the persistent FT in our setting indicates room for systemic improvement. Insights from systems with more robust supports are illustrative. For instance, a comparative study in the United Kingdom–which operates a tax-funded universal healthcare system–found that patients who reported no FT enjoyed a high health utility score of 0.81 (Ngan et al., 2025). This highlights that in our context, policy measures should aim not only at reducing direct out-of-pocket costs but also at strengthening the broader social safety net to preserve patients’ quality of life and financial stability.

### 4.2 Confirmed socioeconomic and clinical drivers of financial toxicity

Our findings reinforce several established predictors of FT. Lower household income was a consistent demographic risk factor, aligning with global evidence that limited resources directly reduce the capacity to absorb out-of-pocket costs (Bygrave et al., 2021). Younger age demonstrated a protective effect, which may be associated with greater ongoing earning potential, familiarity with digital financial tools, and a longer horizon for financial recovery. Conversely, older age was linked to more severe FT. This is consistent with the identified vulnerability of older cancer patients to financial toxicity (Wang L. et al., 2024), which in our context may stem from fixed incomes, accumulated financial depletion, or increased reliance on traditional family support systems (Corrigan et al., 2022). Furthermore, compared to living with a spouse (the reference group), living with children was associated with less severe FT, potentially due to intra-household financial sharing and caregiving support. In contrast, living alone or in other arrangements was a distinct risk factor, underscoring the critical buffering role of stable co-residence, particularly spousal support. Regarding clinical factors, advanced disease stage predicted severe FT, consistent with the shift toward more expensive targeted therapies and immunotherapies (Mollica et al., 2024). A more recent time since diagnosis (<6 months) also had a pronounced effect, capturing the initial “financial shock” phase where costs are sudden, unexpected, and potentially overwhelming (Bell-Brown et al., 2023). This finding identifies a critical window for early intervention by financial navigators or oncology social workers to provide cost transparency, identify resources, and prevent a downward spiral.

### 4.3 Psychological distress: the foremost amplifier of financial toxicity

The most potent predictor in our model was psychological distress (β = −0.594, *p* < 0.001). This aligns with the transactional model of stress and coping, which posits that an individual’s stress response depends on their appraisal of a situation as a threat and their perceived capacity to cope (Lazarus and Folkman, 1984). For liver cancer patients, high treatment costs constitute a major stressor. Those with heightened psychological distress are more likely to appraise this financial strain as an unmanageable threat, triggering anxiety, helplessness, and catastrophic thinking (Yamamoto et al., 2024). This negative cognitive-emotional framework amplifies the subjective experience of FT, patients may report severe financial distress even with moderate objective costs. Our result corroborates a recent meta-analysis confirming a strong association between depression/anxiety and FT across cancer types (Ehsan et al., 2023). Within the Chinese context, this highlights an urgent need for integrated psycho-oncological services. Routine screening for distress and interventions such as cognitive-behavioral therapy–adapted to address cancer-related financial fears–could mitigate this key driver of FT.

### 4.4 Family resilience as a protective buffer

Conversely, family resilience emerged as a significant protective factor (β = 0.207, *p* < 0.001), a finding powerfully supported by the strong negative correlation (*r* = −0.662). This finding is well explained by Walsh’s family resilience framework, which focuses on family strengths rather than deficits (Walsh, 1998). These include collaborative problem-solving (e.g., restructuring budgets, exploring income alternatives), mobilization of social resources (e.g., seeking financial aid from kin networks), and maintaining a shared positive outlook. These processes not only provide tangible financial support but also foster a sense of collective efficacy, which directly cushions the financial distress (Cao et al., 2025). Our study extends existing evidence by quantitatively demonstrating the protective effect of family resilience against FT in liver cancer, a population with high caregiving demands and often catastrophic expenses. This implies that supportive care should evolve to include family-centered interventions, such as training healthcare providers to assess family dynamics and referring families to programs that build communication, problem-solving, and resource-utilization skills.

### 4.5 The counterintuitive role of symptom burden

A notable finding was the greater symptom burden was associated with higher FT scores (indicating less severe FT) (β = 0.159, *p* = 0.002). While counterintuitive–as more symptoms typically incur higher management costs–this paradox may be explained by response shift or competing concerns (Ebbestad et al., 2023). Patients experiencing severe physical suffering (e.g., intractable pain, debilitating fatigue) may cognitively and emotionally prioritize immediate symptom relief and survival above all else (Nipp et al., 2022). Their psychological bandwidth becomes dominated by physical distress, potentially leading them to downplay or report lower levels of financial concern on questionnaires (Su et al., 2022). Furthermore, patients with the highest symptom burdens are often in the palliative phase, where discussions about costly curative-intent therapies may diminish, altering both the financial landscape and its perceived salience. This finding underscores that FT is a subjective construct measured against a patient’s current priorities and echoes reviews noting complex, sometimes inconsistent relationships between FT and symptom burden (Pangestu and Rencz, 2023). Future mixed-methods research is needed to unpack this complexity. This should employ longitudinal designs to track the dynamic relationship between symptoms and FT over the disease trajectory, and integrate qualitative interviews to explore how patients cognitively and emotionally weigh physical suffering against financial concerns in real-time decision-making.

### 4.6 Clinical implications

The findings of this study carry significant and actionable implications for clinical practice in oncology. To effectively mitigate FT, a multi-pronged, integrated approach that concurrently addresses its psychological, familial, and economic dimensions is essential, as outlined below. First, the identification of psychological distress as the strongest independent predictor of FT mandates its systematic integration into routine care. We recommend the routine administration of ultra-brief screening tools (e.g., the PHQ-4) during clinical encounters. Patients identified with significant distress should then have timely access to integrated psycho-oncology services. Second, the robust protective role of family resilience necessitates a deliberate shift toward a family-centered model of care. Clinicians and support staff should be equipped to conduct brief assessments of family functioning. Families identified as needing support should be referred to structured programs designed to build family resilience by enhancing communication, fostering collaborative problem-solving (e.g., budget restructuring, navigating aid resources), and mobilizing social support, thereby empowering the family unit to act as a cohesive buffer against financial adversity. Third, the acute financial shock captured by a more recent time since diagnosis highlights a critical window for pre-emptive intervention. The early integration of financial navigators or oncology social workers into the care team is paramount. At or near diagnosis, these professionals can provide cost transparency, identify insurance and charitable resources, and help formulate financial plans. This proactive guidance can prevent the downstream spiral of debt, treatment non-adherence, and diminished quality of life. Finally, the paradoxical finding that higher symptom burden was associated with less severe FT reports demands heightened clinical vigilance. It suggests that patients experiencing severe physical distress may cognitively deprioritize or underreport financial concerns. Therefore, FT should be assessed systematically in all patients using validated tools like the COST-PROM, rather than relying on spontaneous disclosure, to ensure that financial support is offered equitably and is not overlooked amidst competing clinical priorities. In summary, moving beyond a singular focus on direct costs, a comprehensive strategy to combat FT must weave together routine psychological screening, intentional family support, proactive financial navigation, and systematic FT assessment into the standard oncology workflow.

### 4.7 Study limitations

This study has limitations. Its cross-sectional design precludes causal inference. The sample was from a single center, which may affect generalizability. The use of self-report measures, though standard, is subject to bias. The paradoxical symptom burden-FT relationship warrants in-depth qualitative exploration. Future research should employ longitudinal designs to trace the evolution of FT and its predictors over the cancer trajectory. Intervention studies are urgently needed to test the efficacy of the proposed integrated support models. Furthermore, exploring the dimension-specific effects of family resilience and financial toxicity could yield even more precise intervention targets.

## 5 Conclusion

In conclusion, this study demonstrates that financial toxicity in Chinese liver cancer patients is a multi-dimensional construct, intricately woven from clinical, economic, and psychosocial threads. Family resilience serves as a vital protective buffer, while psychological distress acts as a powerful amplifier. The paradoxical finding regarding symptom burden reveals the complex subjective nature of how patients experience and report financial distress. For the field of supportive care in cancer, these insights are a compelling call to action. Effectively mitigating the pervasive burden of financial toxicity requires a holistic approach that moves beyond mere cost assistance. We must concurrently build the psychological resilience of the patient and the adaptive capacity of the family unit, ensuring that cancer care heals without bankrupting–both financially and emotionally.

## Data Availability

The raw data supporting the conclusions of this article will be made available by the authors, without undue reservation.
